# Circulating extracellular vesicle-microRNAs (EV-miRNAs) in leukemias and related disorders

**DOI:** 10.3389/fmed.2026.1754967

**Published:** 2026-04-28

**Authors:** Tsukuru Umemura, Tatsuki Shibuta, Yukichi Takada

**Affiliations:** 1 Department of Medical Technology and Sciences, Graduate School of International University of Health and Welfare, Ohkawa, Japan; 2 Clinical Laboratory, Kouhoukai Takagi Hospital, Ohkawa, Japan

**Keywords:** biomarker, cell-to-cell communication, extracellular vesicles, leukemia, microRNA

## Abstract

Extracellular vesicles (EVs) are nanoparticles released from all cell types and function as a biological tool for cell-to-cell communication or organs located even in remote parts. The numbers and molecular profiles of circulating EVs, including their cargos, change in response to various physical, chemical, inflammatory, and neoplastic stimuli. Therefore, circulating EVs are expected as the next-generation biomarkers or therapeutic targets. MicroRNAs (miRNAs) are short non-coding RNAs enriched with EVs, which are transported to recipient cells to regulate gene expression. Comprehensive analysis of EV-miRNAs is expected to support precision medicine approaches both as biomarker and therapeutic approaches. This mini review focuses on recent advancements in EVs and miRNAs research in leukemias and related disorders.

## Introduction

All cells release nanoparticles known as extracellular vesicles (EVs), which function as biological tools for cell-to-cell communication between different cell types or organs (1, 2). The profile of circulating EVs changes in response to physiological and pathological processes. EV numbers released from parent cells, their biogenesis pathways, and their cargos vary under various physical and chemical, and inflammatory stimuli, as well as neoplastic transformation (3, 4). Therefore, cells in the whole body share molecular messages via circulating EVs (5–7). MicroRNAs (miRNAs) are short non-coding RNAs (8, 9) and enriched in EVs (1, 2, 10). EV-miRNAs are transported to recipient cells, where they regulate gene expression (5). Thus, EV-mediated transfer of microRNAs is one of the novel genetic mechanisms (1, 5, 11). Comprehensive analysis of EV-miRNAs is expected to develop precision medicine-oriented biomarkers and therapeutic approaches (12–15).

## Biogenesis and subtypes of EVs

EVs are nanoparticles ranging from 50 to 1,000 nm in diameter enveloped in a lipid bilayer membrane that are released from cells and cannot replicate (2). EVs are a heterogenous group according to their biogenesis, particle size, and surface molecules. EVs are categorized as large EVs (size >200 nm), small EVs (<200 nm), and apoptotic bodies (1, 2, 16–19). Large EVs greater than 200 nm in size have been known as microvesicles (MVs), which are produced by outward bulging of the outer membrane of cells. Small EVs under 200 nm in size (also known as exosomes) are produced from the endosome system and fuse with the multivesicular body (MVB) in the cytoplasm and are finally released extracellularly through exocytosis. During the small EV biogenesis, the proteins, nucleic acids, and other biological molecules are selectively loaded into small EVs (1, 3, 7). Apoptotic bodies are produced by segmentation of the cytoplasm as a result of programmed cell death (17). Differences in biological function, release, and uptake, and the kinetics of circulation among each EV subtype are still not fully understood. Differences in cargo between large and small EVs suggest independent biological roles for cell-to-cell communication (20, 21). A recent study using asymmetric flow field-flow fractionation (AF4) discovered non-membranous small nanoparticles <35 nm diameter, termed exomeres, and suggested a different biological role for them as EV subtypes (22).

## Isolation of circulating EVs

**Figure 1:**
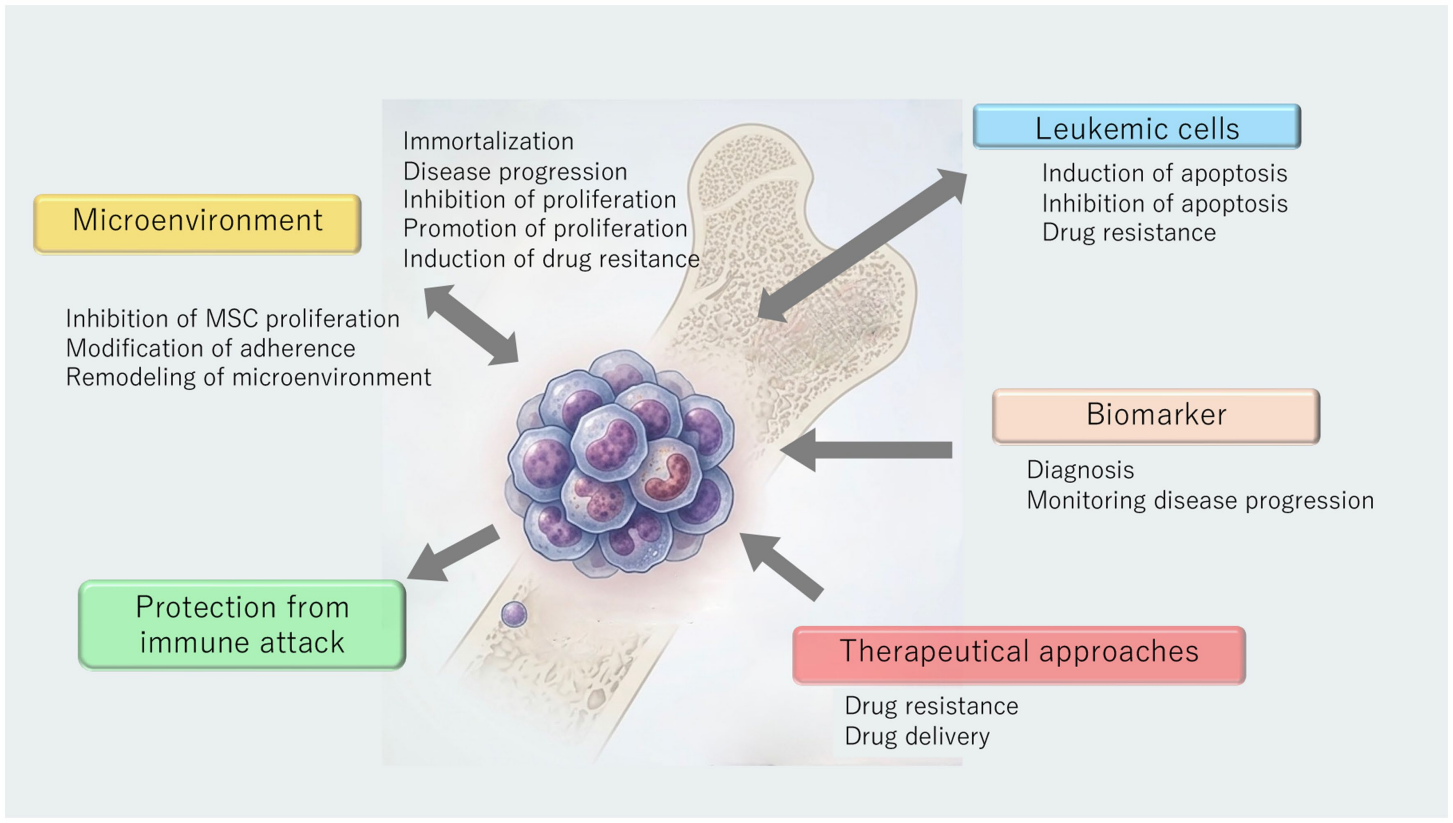
Multiple pathways of EV-mediated cell-to-to-cell communication in leukemia and related disorders. (1) Microenvironment-derived EVs support immortalization, disease progression, cell growth, drug resistance. (2) Leukemia cell-derived EVs induce permissive remodeling of environment. (3) Leukemia cell-derived EVs are horizontally transferred to leukemia cells and modify cell growth or drug resistance. (4) Liquid biopsy of leukemia cell-derived EVs as biomarkers. (5) Possible therapeutical approach.

Circulating EVs are released from all types of cells in the human body, and therefore liquid biopsy is expected to be a relevant tool as a new generation biomarker. However, EVs are a small population in circulating blood. Therefore, standardized purification is essential. Common isolation methods are summarized in Table 1 (2, 21–24). The precipitation method enables high EV recovery. However, the precipitates contain contaminants, including aggregates of proteins and lipoproteins. The ultracentrifugation method, which is one of the conventional technologies, isolates both EVs and heterogeneous EV subtypes. The density gradient ultra-centrifugation methods separate EV subtypes and enable better understanding of biological differences among EV subtypes. When substances that bind to the surface molecules of EVs are available, immune- and affinity-capture methods are effective to isolate specific EV types. Among the new technologies, the microfluidic-based isolation is a powerful approach for isolating various kinds of nanoparticles with high purity. This technology uses microflow systems to isolate EVs by “physical” or “chemical” methods. The advantages of the microfluidic method are small starting volumes, label-free, and the ability to selectively isolate EVs with specific characteristics. This technology can sort EVs of different sizes and shapes and produce single-EV suspensions (25–27) (see Figure 1).

**Table 1:** Commonly used methods for EV isolation.

Methods	Recovery	Specificity
Precipitation	H	L
Differential ultra-centrifugation	M	M
Filter concentration	H	L
Density gradient	M	M
Size-exclusion chromatography	M	M
Immuno-precipitaion	L	H
affinity-precipitaion	M	M
Asymmetric flow field-flow fractionation	L	H

H: high, M: moderate, L: low (2, 21, 23, 24).

## Identification of cellular origin

The surface membrane phenotype and cargos of EVs reflect parent cell types and also the physiological or pathological events (3). Surface molecules of EVs consist of a mixture of ubiquitous components such as tetraspanins (CD63, CD9, and CD81) and lineage-specific cellular components that are available for the identification of cell origin (9, 20, 28, 29). CD63 is a protein enriched in MVB, which produce small EVs, while CD9 and CD81 are generally present on the plasma membrane. Therefore, CD63 is relatively specific to small EVs (exosomes) (2). Common lineage-specific membrane markers are listed in Table 2 (20, 28). One surface molecule is not necessarily specific to a single cell type, as many are expressed in multiple cell lineages. Multiplexed profiling of EVs showed heterogeneity within the same cancer cell line, even when released from a single cell (23, 30). Therefore, it is better to use a set of surface molecules for the identification of the origin of EVs (31, 32). Almost 99.8% of circulating EVs are blood cell-origin in normal subjects (33, 34). Non-blood cell-derived EVs account for only 0.2%, among which fat cell-derived EVs are the most abundant type (81.88%) (34). miRNA analysis also supported these findings (35, 36). Thus, the main sources of circulating EVs are blood cells and adipocytes, suggesting high potential for diagnostic value in hematological malignancies. The flow cytometry detects the surface characteristics of particles passing through the flow tube. Conventional flow cytometry is available for particles larger than 300 nm in diameter (37). Therefore, bead-based flow cytometry is used to detect surface molecules on EVs. If the antibody is available to detect antigens, the immunoaffinity capture method is effective. Koliha et al. reported EV subtypes identified diversity in EV-surface antigens using a multiplex bead-based platform detecting 39 different molecules on the EV surface (38–40). Surface plasmon resonance imaging (SPRi) technology detects interactions between unlabeled paired molecules (ligand/antibody, lectin/lectin-binding molecule, nucleic acid/probe, or drug/target molecule) (41, 42). The paired molecules on the gold-coated surface affect surface plasmon waves, leading to changes in the refractive index. The SPRi analyzes multiple surface molecules at the same time. This study utilized SPRi technology to analyze circulating EVs and found disease-specific profiles of circulating EVs in patients with hypertension and type II diabetes mellitus (43). Neutrophil-derived EVs increased in sepsis (44). Increased CD147-positive EVs were a relevant predictor of myeloma progression or therapeutic effectiveness (45). Activation of platelets induces the release of platelet-derived EVs carrying procoagulant activity in thrombotic diseases (46). Different types of cell lines release the different EV profiles into the culture medium (47). These findings indicate that release of EVs reflects parent cell types and their biological behavior in response to physiological or pathological processes (48, 49). Therefore, analyzing sets of EV surface molecules offers a promising method to detect disease-specific changes in EV profiles in case of hematological malignancies.

**Table 2:** Surface molecules of EVs.

Category	Molecules
Tetraspanins	CD9, CD63, CD37, CD81, CD53
adhesion molecules	Integrin-α, Integrin-β, P-selectin
glycoproteins	Beta-galactosidase, Glycans
Lipids	Cholesterol, Ceramides, Sphingomyelin, Phosphatidylserine, phosphatidylcholine, phosphatidylethanolamine, Phosphatidylinositol, Gangliosides
Receptor proteins	FasL, TNFR, TfR
lineage-specific proteins	cell type-specific CD proteins
Antigen Presenting molecules	MHC Class I, MHC Class II

FasL: Fas ligand; TNFR: tumor necrosis factor receptor; TfR: transfferin receptor (9, 20, 28, 29).

## Kinetics of circulating EVs

Better understanding of the kinetics of circulating EVs is essential for clinical utilization of EVs as biomarkers or therapeutic approaches (4, 46, 48, 49). Surface molecules have essential roles in the uptake of EVs by recipient cells, mainly through interaction between ligand and receptor or antigen–antibody present on the surfaces of EVs and recipient cells. The clearance time of circulating EVs is relatively short. Charoenviriyakul et al. (47) injected fluorescein (gLuc-LA)-labeled exosomes intravenously into five different species of mice and estimated half-times of less than 5 min. Chen et al. (50) injected labeled EVs derived from human embryonic kidney cells (HEK293F) into mice through the tail vein and found that 45.8% of injected EVs were retained in blood for 4 h and there were undetectable levels at 24 h. Yu et al. (51) showed that allo-EVs had a shorter half-life time and that red blood cell-derived EVs had the longest half-life time among blood cell origin EVs. Ngu et al. (52) also pointed out the rapid elimination of milk EVs after oral uptake and suggested their short lifespan in blood is influenced by tissue distribution, excretion in feces and urine, uptake by macrophages, and degradation. Clayton et al. (53) showed that exosomes derived from antigen-presenting cells express CD55 and CD59, which are GPI-anchored complement regulators, protect EVs from attack by complements and permit survival of EVs in the extracellular environment. Kamerkar et al. (54) showed that CD47 molecules on the EV surface prevent phagocytosis by monocytes and macrophages, resulting in prolonged exosome half-life in circulation. On the other hand, phosphatidylserine (PS) is a surface lipid characteristic of apoptotic bodies and is known as the “eat me” signal, which commonly exists on the surface of EVs (48, 55). Thus, the presence of PS on EVs shortens the turnover time of circulating EVs to 5–30 min (47, 50). Therefore, multiple factors determine the profile of circulating EVs, including EV-origin-specific and disease-specific pathophysiology. Understanding the EV kinetics is essential for the development of relevant biomarkers and therapeutic approaches in hematological malignancies.

## microRNAs as EV cargos

Analysis of EV cargos is critical to understand cell-to-cell communication in homeostasis and pathological processes (11, 56). MicroRNAs (miRNAs) are short non-coding RNAs with 19–25 bp length. Mature miRNAs bind to the RNA-induced silencing complex (RISC) and suppress gene expression by digesting or blocking translation of target mRNAs. miRNAs are a small group of total cellular RNA (0.01%) and are located mainly in the MVB within the cytoplasm (9, 56). Sequencing studies of nucleic acid components on EVs showed that miRNAs are abundant RNAs in EVs (5, 7, 57). Almost half of the circulating miRNAs are distributed as EV-miRNAs (7, 12, 58). This localization bias of miRNA enables EVs transport miRNAs as cargos into extracellular spaces and circulation (5). However, regarding miRNA distribution in circulation, there are opposite findings on whether circulating miRNAs are EV- or non-EV (59–61).

## Cargo proteins of EVs

EV-surface or encapsulated proteins showed that the protein profile is specific to each EV subtype (2, 38, 62, 63). Protein components of EVs are categorized into five categories: transmembrane proteins, cytosolic proteins, non-EV co-isolated structures, transmembrane, lipid-bound and soluble proteins, and secreted proteins recovered from EVs (2). These protein components are not necessarily specific for the EV subtypes. Mass spectrophotometric analysis or enzyme-linked immunosorbent assay (ELISA) has shown that protein profiles are characteristic of the lineage or function of parent cells (23, 34, 64). Recent reports showed that the pattern of EV surface molecules is modified through binding to molecules present in plasma (named as biomolecular corona or EV corona) (65, 66). More understanding of the EV corona may contribute to the development of more relevant biomarkers and therapies.

## EV miRNA as biomarkers of hematological malignancies

Increased particle numbers of circulating EVs were reported in hematological malignancies: chronic lymphocytic leukemia (CLL), diffuse large B-cell non-Hodgkin lymphoma (DLBC-NHL), follicular non-Hodgkin lymphoma (F-NHL), and primary myelofibrosis (PMF) compared to normal subjects (67). Feng et al. (68) used miRNA microarray analysis to observe the difference between miRNA profiles of the K562 leukemic cell line and released EVs. In exosomes, 49 miRNAs were upregulated in comparison with K562 cells. These findings suggested that leukemic cell-derived EVs carry distinct miRNA profiles. Leukemia-cell- or microenvironment-derived EV-miRNAs have been reported as crucial molecules in the pathophysiology of leukemia and related diseases. Understanding the biological significance of EV-miRNAs is required for developing precision medicine-oriented biomarkers. The mechanism of cell-to-cell communication in leukemia can be categorized as microenvironment-to-leukemic cells, leukemic-cells-to-microenvironment, and leukemic-cells-to-leukemic cells (Table 3), which are potent candidates for relevant biomarkers or therapeutic approaches.

**Table 3:** Rev. 1: EV-miRNAs and biological effects in leukemias and related disorders.

Categories of miRNAs functions	Diseases	EV-miRNAs	References
Interaction with microenvironment etc.	AML	miR-222-3p	Zhang et al. (2020) (69)
		miR-23b-5p	Cheng et al. (2021) (70)
		miR-139-5p	Wang (2024) (71)
		miR-140-3p	Li et al. (2025) (72)
		miR-145a-5p	Chen et al. (2022) (73)
		miR-196a-5p	Fan et al. (2025) (75)
		miR-711	Jiang et al. (2020) (80)
		miRNA profile	Li et al. (2022) (83)
		miR-34c-5p	Peng et al. (2018) (107)
		miR-26a-5p	Ji et al. (2021) (108)
	ALL	miR-34a-5p	Taverna et al. (2021) (109)
	CML	miR-92a-3p	Wan et al. (2019) (81)
		miR-140-3p	Asano et al. (2017) (93)
		miR-126	Taverna et al. (2014) (109)
		miR-21	Taverna et al. (2016) (109)
		miR-320	Gao et al. (2019) (110)
		miR-126	Zhang et al. (2018) (111)
	CLL	miR-155-5p, miR-146a-5p, miR-132-3p	Dubois et al. (2024) (87)
		miR-146a	Yang et al. (2020) (84)
		miR-202-3p	Farahani et al. (2015) (112)
		miR-155	Bruns et al. (2017) (113)
	Myeloma	miR-16	Khalife et al. (2019) (114)
	Miscellaneous	miR-29b	Yoon et al. (2025) (76)
		miR-7977	Horiguchi et al. (2016) (115)
Proliferation & differentiation	AML	miR-4498, miR-3156-5p, niR-23a-5p, miR-19a-3p, miR-181b-5p	Kariminejad-Farsangi et al. (2025) (94)
		miR-339-5p	Charoensedtasin et al. (2024) (100)
		miR-125b	Chen et al. (2022) (116)
		miR-221-3p	Li et al. (2024) (117)
		miR-34c-5p	Wen et al. (2023) (118)
	ALL	miR-29b-3p	Zhou et al. (2023) (74)
		miR-326	Dashti et al. (2025) (119)
	CML	miR-21	Taverna et al. (2015) (120)
Disease progression	AML	miR-570-3p	Bi et al. (2021) (90)
		miR-10b	Fang et al. (2020) (88)
		miR-125b	Jiang et al. (2018) (89)
	ALL	miR-181b-5p	Yan et al. (2021) (121)
	CML	miR-494	Shibuta et al. (2022) (91)
		miR130b-3p	Chai et al. (2023) (122)
	Myeloma	miR-140-3p, miR-584-5p, miR-191-5p, miR-143-3p	Gregorova et al. (2025) (92)
Biomarker	AML	miRNA profiles	Feng et al. (2013) (68)
	ALL	miR-1290	Zavala-Reyes et al. (2025) (123)
	CLL	miR-363, miR-16	Alharthi et al. (2018) (124)
Drug resistance	AML	miR-20b-5p	Huang et al. (2025) (95)
		miR-10a	Wu et al. (2022) (99)
	CML	miR-548b-3p	Tang et al. (2025) (98)
		EV miRNA profile	Navakanitworakul et al. (2025) (101)
		miR-629-5p	Jiang et al. (2024) (96)
		miR-125b-5p, miR-99a-5p, miR-210-3p, miR-193b-3p	Karabay et al. (2024) (97)
		miR-365	Min et al. (2018) (125)
	CLL	miR-328	Dong et al. (2019) (126)
	MDS	miR-4755-5p	Lei et al. (2023) (127)

Hematopoietic stromal cells support the maintenance, proliferation, and differentiation of normal stem cells. In leukemia and related diseases, this hematopoietic network changes. Growth inhibition of leukemic cells was reported in several studies through miR-223-3p/IRF2/INPP4B (69), miR-23b-5p/TRIM14 (70), miR-139-5p/*β*-catenin/Bax (71), miR-140-3p/SUZ12 (72), miR-145-5p/USP6/GLS1 promotes imatinib-induced apoptosis (73), and miR-29b-3p/GDF15/MAPK (74). On the other hand, other studies showed promotion of leukemic cell growth by the miR-196a-5p/ferroptosis (75) or miR-29b axis (76). These findings showed the multiple pathways through which EV-miRNAs act in cell-to-cell communication from the microenvironment to leukemia cells.

Leukemic cell-derived EVs induced changes in the microenvironment that supported leukemic cell survival (77–86). EV-miR-711 from the K562 cell line suppressed the adhesiveness of bone marrow mesenchymal stem cells, creating a favorable microenvironment for leukemic cell proliferation (80). Suppression of adipogenesis (81), enhancement of angiogenesis (82, 83), or induction of cancer-associated fibroblasts (84) were also reported as the effects of leukemic cell-derived EV-miRNAs, which create permissive changes for leukemic cell growth. *In vivo* experiments also showed that remodeling of the stem cell niche by leukemic cell-derived EVs made the microenvironment permissive for leukemic cell survival (85). Leukemic cell-derived exosomes suppress normal hematopoiesis via miR-155 and miR-150 targeting c-MYB (86) or promote disease progression via miR-155-5p/miR-146a-5p/miR-132-3p (87). Therefore, leukemia cell-to-microenvironment communication is also an important pathophysiological mechanism.

Several EV-miRNAs play an important role in disease progression. Patients with acute myelogenous leukemia (AML) who showed high levels of miR-10b in circulating EV had shorter survival in a cohort of 95 *de novo* AML (88). EV miR-125b (89) and miR-570-3p (90) correlate with AML progression. Shibuta et al. (91) reported higher levels of miR-494 in circulating EVs obtained from the accelerated phase of chronic myelogenous leukemia (CML) than in the chronic phase. A crucial role of EV-miR-494 in CML progression and its possible use as a biomarker for early diagnosis of the accelerated phase was suggested. Gregorova et al. (92) used miRNA sequencing to analyze changes of EV-miRNAs in patients with multiple myeloma, extramedullary disease, and plasma cell leukemia and showed a possible contribution of a dysregulated EV-miRNA profile in patients with plasma cell malignancy. Significant elevation of miR-140-3p levels was observed in CML patients with musculoskeletal pain that appeared after stopping tyrosine kinase inhibitor administration (93). These findings clearly suggest the clinical relevance of EV-miRNAs as biomarkers for diagnosis, pathophysiology, disease progression, and monitoring of minimal residual disease (MRD).

Overcoming drug resistance is one of the major challenges in the treatment of hematological malignancies. Inhibition of EV release with imipramine, which blocks EV formation, resulted in antileukemic effects on the NB4 cell line established from acute promyelocytic leukemia. The profiling of miRNAs also showed changes in miRNA profiles that target genes involved in cancer-related pathways (94). Many studies have indicated the contribution of miRNAs to drug resistance: miR-20b-5p/MASTL/PI3K/AKT axis (95), miR-629-5p/PI3K/AKT/mTOR axis (96), 125-5p and miR-99a-5p profile change (97), and circ_0058493/miR-548b-3p/U25URP axis (98). Wu et al. showed that bone marrow mesenchymal stem cell-derived exosomes transferred miR-10a and enhanced chemoresistance of acute myeloid leukemia cells (99), suggesting a contribution of microenvironmental cells to drug resistance. An anti-leukemia effect of PG2 was achieved by leukemic cell-derived EVs through apoptosis induction using the CDK2/miRNA-339-5p caspase-3 axis (100), indicating a possible approach to interrupt EV-mediated chemoresistance.

Current limitations are that the accumulating observations are still based on analysis of a single miRNA and that the obtained data lack relevant consistency. The reason for these confusing results might be multiple links between miRNAs and target genes. Recent studies have used the analysis of multiple miRNAs in EVs as disease biomarkers. Comprehensive profile analysis of miRNAs is expected to be a more relevant approach (68, 91, 94, 97, 101).

## Conclusion and limitation

EV-mediated transfer of miRNAs is a novel genetic mechanisms of cell-to-cell communication. Many pathological dysregulated EV-miRNAs in hematological malignancies have been reported, including transfer of miRNAs from microenvironment-to-leukemic cell, leukemic cell-to-microenvironment, and leukemic cell-to-leukemic cell (horizontal transfer). The clinical outcomes include the development of relevant biomarkers and therapeutic approaches (15, 102–104). Therefore, analysis of circulating EVs and EV-miRNAs using liquid biopsy is promising for developing relevant biomarkers. Differences in biological functions among EV subtypes are not yet fully unveiled. The standardized isolation method of the EV subtypes may improve the understanding of EV-miRNA biology in homeostasis and disease. Based on the knowledge of a wide variety of miRNA target genes and different profiles of EV-miRNAs in diseases, a more comprehensive analysis of EV-miRNA profiles, such as a report done by Li et al. (83), since each miRNA has hundreds of target mRNAs, and one mRNA has multiple miRNA binding sites (8, 9, 105, 106). Principle component analysis, predictions of target mRNA, and downstream pathways may promote a better understanding of cell-to-cell communication through EV-miRNAs. Overcoming these challenges in EV-miRNA biology is crucial for developing relevant disease biomarkers and therapeutic approaches.
